# Where Cholera Prevails

**Published:** 1849-02

**Authors:** 


### WHERE CHOLERA PREVAILS.

Dr. Jackson, of Boston, as will be seen under our head of Selections, has remarked that cholera does not prevail in Granite regions. We sometime ago called the attention of our readers to the fact, that it was in places where Blue Limestone is the surface rock, that cholera was most fatal when it invaded our country before. If the disease should again become epidemic, we shall have opportunities of observing how far it follows the same route, and conforms to the same laws.

